# ATP Induces Interleukin-8, Intracellular Calcium Release, and ERK1/2 Phosphorylation in Bovine Endometrial Cells, Partially through P2Y Receptors

**DOI:** 10.3390/ani13050841

**Published:** 2023-02-25

**Authors:** Noemí Gutierrez, Stefanie Teuber, Pablo Alarcon, Rafael A. Burgos, María A. Hidalgo

**Affiliations:** Institute of Pharmacology and Morphophysiology, Faculty of Veterinary Sciences, Universidad Austral de Chile, Valdivia 5090000, Chile

**Keywords:** ATP, calcium, purinergic receptors, endometrial cell

## Abstract

**Simple Summary:**

Bovine uterine infections are common in the postpartum period and are associated with economic losses in dairy herding. Adequate immune function is key to preventing disease. Adenosine triphosphate (ATP) is a known activator of the inflammatory response, and can be produced in the endometrial microenvironment. In this study, we observed that ATP increased the proinflammatory responses in bovine endometrial cells, such as release of chemokine interleukin-8 (IL-8), intracellular calcium mobilization, and ERK1/2 phosphorylation. Additionally, we demonstrated the presence of a subtype of purinergic receptors (P2Y), specifically, high levels of P2Y1 and P2Y2 receptors. The inhibition of P2Y receptors reduced the proinflammatory responses induced by ATP. The results suggest that P2Y receptors play a role in endometrial inflammatory activation, which could be useful as a therapeutic strategy to regulate uterine inflammation through the modulation of P2Y receptors.

**Abstract:**

The bovine endometrium has an important defensive role in the postpartum period that acts when an inflammatory process associated with tissue damage or infection by bacteria is produced. Endometrial cells release cytokines and chemokines that recruit inflammatory cells, which release danger-associated molecular patterns (DAMPs), such as adenosine triphosphate (ATP), and initiate and regulate the inflammatory response. However, the role of ATP in bovine endometrial cells is unclear. The aim of this study was to determine the effect of ATP on interleukin-8 (IL-8) release, intracellular calcium mobilization, ERK1/2 phosphorylation, and the role of P2Y receptors, in bovine endometrial cells. Bovine endometrial (BEND) cells were incubated with ATP and the IL-8 release was determined by the ELISA assay. ATP of 50 and 100 μM significantly increased IL-8 released in BEND cells (50 μM: 23.16 ± 3.82 pg/mL, *p* = 0.0018; 100 μM: 30.14 ± 7.43 pg/mL, *p* = 0.0004). ATP (50 μM) also induced rapid intracellular calcium mobilization in Fura-2AM-loaded BEND cells, as well as ERK1/2 phosphorylation (ratio 1.1 ± 0.04, *p* = 0.0049). Suramin (50 μM), a pan-antagonist of P2Y receptors, partially reduced the intracellular calcium mobilization, ERK1/2 phosphorylation (ratio 0.83 ± 0.08, *p* = 0.045), and IL-8 release (9.67 ± 0.02 pg/mL, *p* = 0.014) induced by ATP. Finally, BEND cells expressed higher mRNA levels of P2Y1 and P2Y2 purinergic subtype receptors, and lower levels of P2Y11 and P2Y12 receptors, as determined by RT-qPCR. In conclusion, these results showed that ATP activates pro-inflammatory responses in BEND cells, which are partially mediated via P2Y receptors, and BEND cells express the mRNA of subtypes of P2Y receptors, which could have a key role in bovine endometrial inflammation.

## 1. Introduction

Bovine uterine infections are a common health problem in the postpartum period and lead to economic losses due to treatment costs, lost milk production, and infertility [1,2]. The endometrium, as well as being the site of embryo implantation and responsible for the regulation of the estrous cycle through the release of prostaglandins, has a defensive role against infection through classical innate immune mechanisms [3,4,5,6,7]. After parturition, all mammals can develop an inflammatory process associated with tissue damage or infection by bacteria [6]. Diverse cytokines and chemokines are produced by endometrial cells in response to tissue damage or infection, and an influx of inflammatory cells such as polymorphonuclear (PMN) are recruited into the endometrium, all of which favor an inflammatory environment [2,8,9]. Interleukin-8 (IL-8), a strongly attractant chemokine for neutrophils, is released by bovine endometrial cells and produced at high levels in the endometrium in the postpartum period, and in cows with subclinical or clinical endometritis [10,11,12]. PMNs also contribute to inflammation with the release of pro-inflammatory mediators such as adenosine triphosphate (ATP) [13], which acts as a danger-associated molecular pattern (DAMP), and initiates and regulates the inflammatory response along with other damage signals [14,15]. 

ATP binds to purinergic receptors of type P2. There are 2 subtypes of P2 receptors: P2X and P2Y receptors. P2Y receptors are G protein-coupled receptors, and eight receptor subtypes have been identified in humans and rodents: P2Y1, P2Y2, P2Y4, P2Y6, P2Y11, P2Y12, P2Y13, and P2Y14 [14,15,16]. In bovines, P2Y receptors have been identified in neutrophils, retinal endothelial cells, chromaffin cells, chondrocytes, and synoviocites [17,18,19]. However, its presence in bovine endometrial cells is unknown. Some studies have reported the presence of P2Y and P2X subtype receptors in the human endometrium [20,21,22,23,24] and the rat uterine epithelium [25].

After binding to its receptors, ATP activates different signaling pathways. ATP induced intracellular calcium mobilization in human endometrial cancer cells [24]. In human endometrial stromal cells, ATP activates the pathway PLC/PKC/ERK [22]. ERK1/2 MAPK is a signaling pathway involved in the activation of transcription factors, such as the nuclear factor-kappaB (NF-κB), and the IL-8 gene has NF-κB binding sites in its promoter region that regulate its expression [26,27]. However, little evidence exists regarding the role of ATP in endometrial innate immunity. Another nucleotide, UDP-glucose, stimulates IL-8 production in human endometrial epithelial cells, and neutrophil chemotaxis in an IL-8-dependent manner [21].

We hypothesized that ATP activates inflammatory responses such as intracellular calcium release, ERK1/2 phosphorylation, and IL-8 production in bovine endometrial cells, and these responses could be mediated via P2Y subtype receptors. 

## 2. Materials and Methods

### 2.1. Cell Culture

Bovine endometrial (BEND) cells (ATCC^®^ CRL2398™) were obtained from ATCC (Manassas, VA, USA). BEND cells, a cell line derived from the uterine endometrium of a cow on d14 of the estrous cycle [28], were cultured according to the protocol recommended by the manufacturer and as described by Valenzuela et al. [29]. Briefly, a 1:1 mixture of Ham’s F12 and Eagle’s Minimal Essential medium with Earle’s BSS (D-valine modification) containing 1.5 mM L-glutamine adjusted to contain 1.5 g/L sodium bicarbonate (Sigma-Aldrich Co, St Louis, MO, USA) was used. The culture medium was supplemented with 0.034 g/L D-valine (Sigma-Aldrich Co, St Louis, MO, USA), 10% heat-inactivated fetal bovine serum (BioWest, Kansas City, MO, USA), 10% heat-inactivated horse serum (Gibco-Brl, Grand Island, NY, USA), 100 U/mL penicillin, and 100 μg/mL streptomycin (Hyclone, Logan, UT, USA). Before each experiment, the culture medium was changed into a serum-reduced culture medium (2% serum) in the absence of antibiotics, and the cells were maintained for two hours.

### 2.2. IL-8 ELISA Assay

BEND cells (n = 3 independent experiments) were cultured with vehicle (PBS) or different concentrations of ATP (0.1, 1, 10, 50, or 100 μM) (Cayman Chemical, Ann Arbor, MO, USA) for 24 h at 37 °C. LPS (500 ng/mL) (Escherichia coli O111:B4, Sigma-Aldrich, Saint Louis, MO, USA) was used as a positive control for the IL-8 assay. For specific experiments, BEND cells (n = 3 independent experiments) were cultured with 50 μM suramin for 15 min and then 50 μM ATP was added, followed by incubation for 24 h at 37 °C. IL-8 in the supernatants was assessed in duplicate using the Bovine IL-8 (CXCL8) ELISA BASIC Kit (MABTECH AB, Nacka Strand, Sweden), according to the instructions provided by the manufacturer. 

### 2.3. Intracellular Calcium Mobilization

BEND cells (1 × 10^7^ cells/mL) (n = 3 independent experiments) were trypsinized (0.25% trypsin/EDTA), washed in Hank’s balanced salt solution (HBSS) (136 mM NaCl, 5 mM KCl, 0.9 mM CaCl_2_, 0.4 mM KH_2_PO_4_, 0.3 mM NaH_2_PO_4_, 6 mM glucose, pH 7.4), and loaded with Fura-2-AM, according to the method described by Valenzuela et al. [29]. Briefly, BEND cells were incubated with 2.5 μM Fura-2-AM (Invitrogen, Carlsbad, CA, USA) for 30 min at 37 °C in the dark, and then two washes of HBSS were performed. Finally, the cells were suspended in HBSS containing 1 μM probenecid (Invitrogen, Carlsbad, CA, USA). Each experiment was performed in a cuvette maintained at 37 °C with constant stirring in an LS55 spectrofluorometer (PerkinElmer Life Science, Waltham, MA, USA). After 60 s of basal measurement, every 0.2 s at 509 nm emission and 340/380 nm dual-wavelength excitation, vehicle (HBSS) or ATP (0.5, 1 or 50 μM) was added, and the signal registered for 240 s. In another set of experiments, the cells were incubated with vehicle (DMSO 0.1%) or suramin (10, 50, or 100 μM) for 15 min at 37 °C, and then the fluorescence intensity was detected for 60 s of basal measurement. ATP (50 μM ) was then added, and the signal registered for 140 s. 

### 2.4. Immunoblot

BEND cells cultured in a 60 mm plate (n = 3 independent experiments) were treated with vehicle (0.1 % DMSO) or 50 μM suramin for 15 min, and then 50 μM ATP (or vehicle PBS) was added, followed by incubation for 5 min at 37 °C. The cells were lysed according to Manosalva et al. [30,31]. Briefly, cells were incubated with a lysis buffer (50 mM Tris–HCl, pH 7.4, 150 mM NaCl, 1 mM EDTA, 1 mM EGTA, 10 mg/mL aprotinin, 10 mg/mL leupeptin, 5 mM phenylmethylsulfonyl fluoride (PMSF), and 1 mM DTT) on ice, and centrifuged at 18,000× *g* for 20 min at 4 °C. Protein concentration was determined using the Bradford method (Sigma-Aldrich, Saint Louis, MO, USA). Fifty micrograms of protein were used on 10% SDS–PAGE gels. The proteins were transferred to PVDF membranes, and the immunoblot was performed according to the protocol recommended by the manufacturer of the antibody phospho-ERK1/2 (Cell Signaling, Beverly, MA, USA). The antibody against phospho-ERK1/2 was incubated overnight at 4 °C. Then, the membranes were washed and incubated with an HRP-conjugated secondary antibody (LI-COR, Lincoln, NE, USA). The bands of phospho-ERK1/2 were visualized using an Odyssey Fc infrared/chemiluminescent detection system (LI-COR Biosciences). The primary antibody was stripped, and the membranes were incubated with total anti-ERK1/2 antibody (Cell Signaling, Beverly, MA, USA) for 2 h at room temperature. A HRP-conjugated secondary antibody was used and the signal was detected as described above for phospho-ERK1/2 antibody. The band intensity was measured using image Studio Lite v5.2 software (LI-COR Biosciences). The results are shown in a representative image of three experiments, and the band intensity of phspho-ERK1/2 and ERK1/2 is shown in a bar graph as the ratio phspho-ERK1/2/ERK1/2.

### 2.5. Real Time-qPCR

BEND cells (n = 3) grown in 100 mm-plates were used for total RNA isolation, using the EZNA™ Total RNA Isolation Kit (Omega Bio-Tek, Norcross, GA, USA). To ensure removal of genomic DNA, all samples of total RNA were treated with DNase. For cDNA synthesis, 250 ng of total RNA were subjected to reverse transcription with Affinity Script RT (Agilent, Santa Clara, CA, USA), and then real-time PCR was performed in duplicate using the Brilliant II SYBRGreen qPCR K it (Agilent) with primers specific for bovine P2Y receptors subtype and the housekeeping ribonucleoprotein S9 (RSP9) (Table 1) [17,32]. The following conditions were used: 95 °C for 3 min, 40 cycles of 10 s at 95 °C, and 60 s at 60 °C. Then, three additional steps (dissociation curve) were performed: 95 °C for 15 s, 60 °C for 60 s, and 95 °C for 15 s. The post-PCR melting curves confirmed the specificity of the single-target amplification. The efficiency of the reaction was determined by the StepOne software and ranged between 95 and 110% for the different primers. The abundance of each gene was calculated relative to RSP9 mRNA using the 2^−ΔΔCt^ method [33]. The formula used is: relative abundance = 2 ^(−ΔCt)^, where ΔCt is calculated as the difference between the Ct of each P2YR (P2Y1R, P2Y2R, P2Y4R, P2Y6R, P2Y11R, P2Y12R, or P2Y13R) and RSP9 [32,34]. The results are shown in a bar graph, as the relative expression of mRNA of each P2Y receptor. 

### 2.6. Viability Assay

BEND cells (n = 3 independent experiments) were cultured with vehicle (0.1% DMSO) or suramin (1, 10, 100, or 300 μM) for 30 min or 24 h at 37 °C, and cell viability was assessed, in duplicate, using the propidium iodide method as described by Valenzuela et al. [29]. Briefly, 5 μM propidium iodide (Molecular Probes, Invitrogen, Carlsbad, CA, USA) were added and incubation was implemented for 15 min at 37 °C. A positive control for cell death was performed in cells incubated with 0.2% triton X-100 at 37 °C for 40 min. The propidium iodide signal was detected with a fluorescence multiplate reader (Varioskan^®^ Flash, Thermo Fisher Scientific, Waltham, MA, USA) at 530 nm excitation/620 nm emission. The results are shown as % of cell viability.

### 2.7. Statistical Analysis

The experiments (three independent experiments) were analyzed with a one-way analysis of variance and Dunnett’s multiple comparison test (compared with the control or basal), performed with a significance level of 5%. An unpaired t-test was performed in selected groups of IL-8 assay and immunoblot; comparisons between groups are shown in each graph with square brackets and significance level. All analyses were performed with the GraphPad Prism 8.0 program. The results are shown as bar graphs of the mean ± SEM.

## 3. Results and Discussion

### 3.1. ATP Increased IL-8 Levels

**Figure 1:**
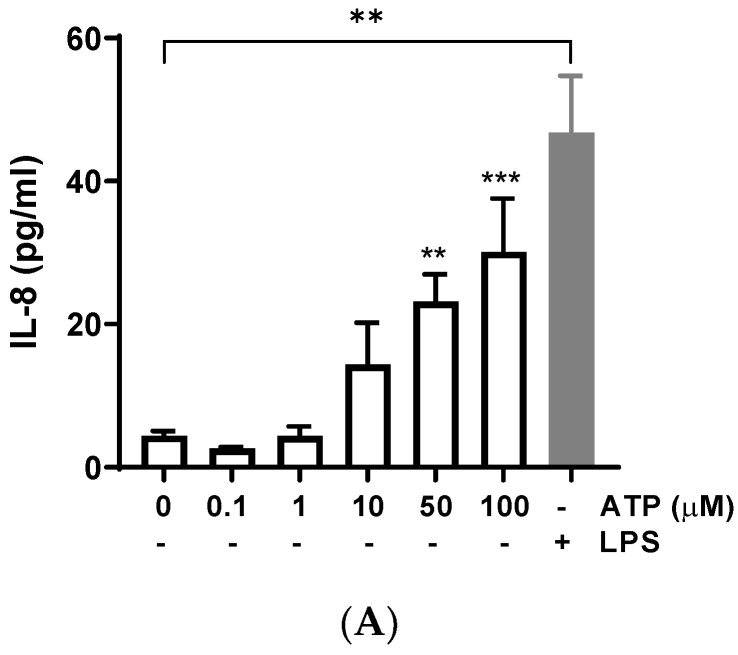

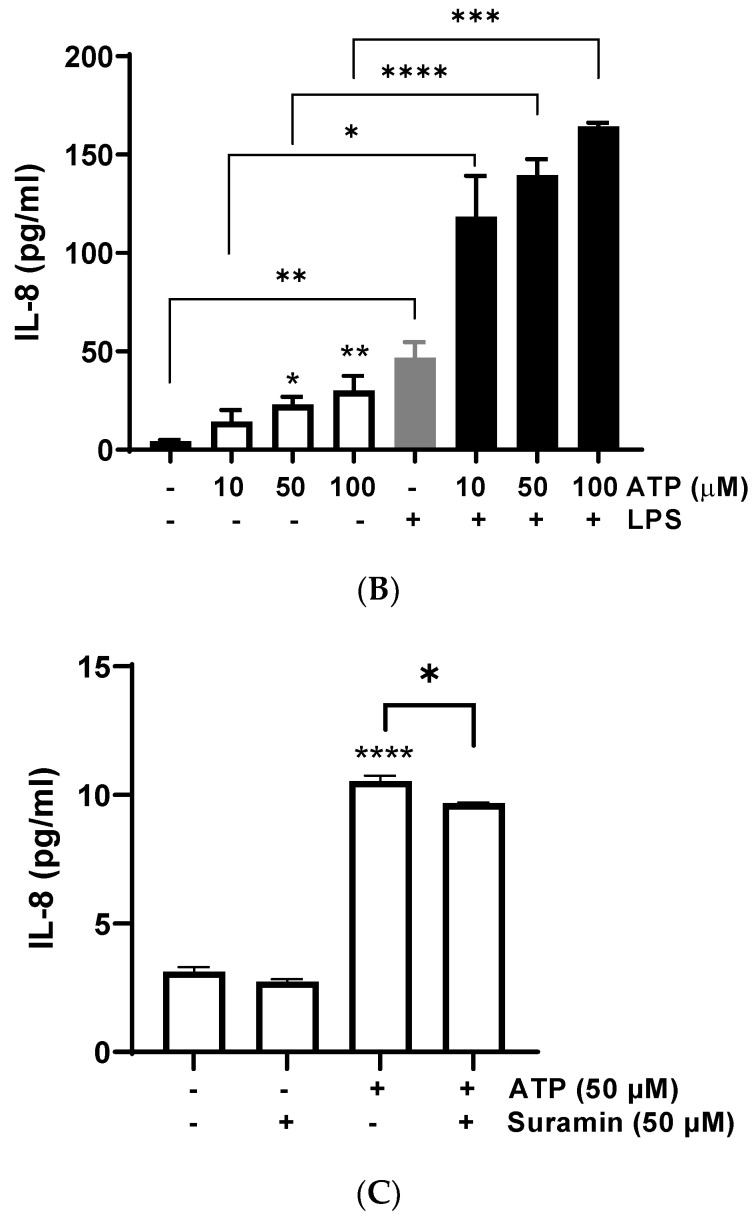
ATP increases interleukin-8 (IL-8) production in BEND cells. (**A**) BEND cells were treated with vehicle (PBS) or different concentrations of ATP (0.1, 1, 10, 50, and 100 μM) for 24 h, and IL-8 production was assessed in the culture medium by ELISA assay. LPS (500 ng/mL) was used as a positive control. (**B**) BEND cells were incubated with vehicle (PBS) or ATP (10, 50, and 100 μM) without or with LPS (500 ng/mL) for 24 h, and IL-8 production was assessed in the culture medium by ELISA assay. (**C**) BEND cells were incubated with vehicle (DMSO 0.1%) or suramin (50 μM) for 15 min, and then ATP (50 μM) was added for 24 h, and IL-8 production was determined by ELISA assay. n = 3 independent experiments. * *p* < 0.05, ** *p* < 0.01, *** *p* < 0.001, **** *p* < 0.0001.

Bovine endometrial cells were treated with different concentrations of ATP for 24 h, and IL-8 release was determined in the supernatant. ATP (50 and 100 μM) significantly increased the production of IL-8 compared with the control (vehicle) (Figure 1A) (50 μM: 23.16 ± 3.82 pg/mL, *p* = 0.0018; 100 μM: 30.14 ± 7.43 pg/mL, *p* = 0.0004); similarly, the positive control LPS induced IL-8 release (*p* < 0.01), as has been described in several studies [35,36,37]. The treatment of BEND cells with ATP plus LPS showed an additive effect on IL-8 release (Figure 1B), for each concentration of ATP assessed.

The contribution of different cells producing ATP into the uterine environment could have important consequences, including reducing bovine reproductive performance. ATP is released from inflammatory cells in response to bacterial peptides and inflammatory mediators [13]. Likewise, ATP is released from endometrial epithelial cells, uterine blood vessels, and nerves innervating the uterus [38]. ATP regulates classical endometrial functions such as implantation of the fertilized oocyte, endometrial fluid functionality, cell proliferation and differentiation in post-partum, apoptosis, and sperm functionality [38]. However, few studies have evidenced the role of ATP in the endometrial inflammatory response. We observed a significant increase of IL-8 production in BEND cells stimulated with ATP, and in BEND cells treated with ATP plus LPS. High levels of IL-8 in the uterus promote an exacerbated inflammatory response, because more immune cells could be attracted. Inflammatory cells, such as neutrophils and macrophages, produce IL-8 under activation by stimuli associated with infection by bacteria or tissue damage [27,30,39,40,41]; therefore, the control of the uterine immune response is crucial for the resolution of inflammation. 

To assess a possible role of purinergic receptors in the increase of IL-8 induced by ATP, BEND cells were incubated with the P2Y pan-antagonist suramin for 15 min. Then, ATP was added and incubation carried out for 24 h. The IL-8 production was partially yet significantly reduced by suramin (Figure 1C) (*p* < 0.05), suggesting a partial role of P2Y receptors in this response. The partial suramin-induced reduction of IL-8 can be explained because ATP does not only activate P2Y receptors. ATP also activates P2X purinergic receptors and can be hydrolyzed into adenosine and activate different signaling pathways, which may contribute to gene expression [14]. The participation of P2Y receptors in endometrial function has been previously suggested in other species. In human endometrial cells, the inhibition of the P2 receptor attenuated the ATP-induced activation of MAPK [22]. Additionally, suramin blocked the stimulatory effect of ATP on prostaglandin production in a guinea pig’s uterus [42]. Thus, our results with suramin support the participation of P2Y receptors in IL-8 production in bovine endometrial cells.

### 3.2. ATP Induces Intracellular Calcium Mobilization and ERK1/2 Phosphorylation

**Figure 2:**
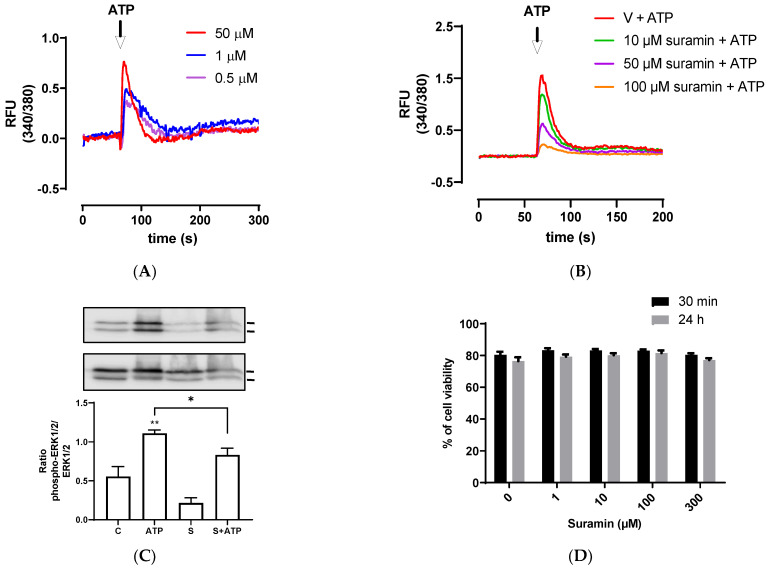
ATP increases intracellular calcium mobilization and ERK1/2 phosphorylation. (**A**) Fura 2AM-loaded BEND cells were treated with vehicle (PBS) and basal fluorescence was registered for 60 s; then, different concentrations of ATP (0.1, 1, or 50 μM) were added and the fluorescence was determined for 240 s. RFU = relative fluorescence units. (**B**) Fura 2AM-loaded BEND cells were incubated with vehicle (0.1% DMSO) or suramin (10, 50, or 100 μM) for 15 min. Then, the basal fluorescence was registered for 60 s and ATP (50 μM) was added, and the fluorescence registered for 140 s. V = vehicle. (**C**) BEND cells were incubated with vehicle (0.1% DMSO) or suramin (50 μM) for 15 min, and then vehicle (PBS) or ATP (50 μM) was added, and incubation was carried out for 5 min. Total proteins were analyzed by immunoblot with antibodies against phospho-ERK1/2 and total ERK1/2. c = control (0.1% DMSO). S = Suramin. Bands’ intensities are shown as the ratio phospho-ERK1/2/ERK1/2 in the bar graph. (**D**) BEND cells were incubated with vehicle (0.1% DMSO) or suramin (1, 10, 100, or 300 μM) for 30 min or 24 h, and the cellular viability was measured by propidium iodide assay. * *p* < 0.05, ** *p* < 0.01 compared with the control. Images are representative of three independents experiments.

Since P2Y receptors are G-protein coupled receptors that activate second messengers, such as inositol triphosphate (IP3), and then lead to an fast increase of intracellular calcium released from the endoplasmic reticulum [43], we assessed intracellular calcium mobilization induced by different ATP concentrations. BEND cells rapidly increased intracellular calcium mobilization after ATP addition, ranging between 0.5–50 μM (Figure 2A). To assess the participation of P2Y receptors, BEND cells were incubated with suramin for 15 min. Then, ATP was added and intracellular calcium was measured. Suramin reduced the intracellular calcium mobilization induced by ATP (Figure 2B), suggesting the participation of P2Y receptors in the effect stimulated by ATP.

Calcium has diverse functions in the uterus, such as the production of developing follicle hormones, estrogen synthesis, and contraction and relaxation [44,45]. A study in bovines showed that the treatment of endometrial explant with the calcium ionophore A23187 increased the prostaglandin F2α synthesis induced by estradiol, thus demonstrating the role of calcium in hormone synthesis [46]. At a cellular level, calcium regulates different cellular processes, such as cell proliferation and apoptosis, gene expression, and inflammasome activation [47,48]. In bovine endometrial cells, it was shown that infection with *E. coli* increased the cytoplasmatic calcium, and the treatment with intracellular calcium chelators reduced the expression of IL-1β and IL-18, proposing the importance of calcium in pro-inflammatory gene expression [49]. In addition, prostaglandin F2α induced IL-8 expression in endometrial adenocarcinoma cells via the PKC/calcium/NFAT signaling pathway [50]. 

The reduction of intracellular calcium mobilization in BEND cells treated with suramin and ATP suggest the participation of P2Y subtype receptors in this response. Several previous studies done with suramin showed a reduction in ATP-induced calcium response in different cell types, such as human endocervical cell line, bovine trophoblast cell line, and canine macrophage cell line, and suggested the participation of P2 receptors in the rise of calcium [51,52,53].

Calcium is a second messenger that activates intracellular signaling pathways, such as MAPK in human endometrial cells [54,55]. Therefore, we assessed whether ATP could induce ERK1/2 phosphorylation in BEND cells. We observed that 50 μM ATP incubated for 5 min significantly increased ERK1/2 phosphorylation (*p* < 0.01) compared with the control, and this effect was partially but significantly reduced by the antagonist suramin (*p* < 0.05) (Figure 2C). The band sizes detected in all experiments correspond to the size expected for ERK1/2 (44 and 42 kDa). Antibody against total ERK1/2 (independent of the phosphorylation status) was used as loading control, therefore, differences in the amount of protein loaded in each line of the gel do not interfere in the result, because the bands of each line were normalized to total ERK. In human endometrial stromal cells, it was shown for first time that ATP activates ERK1/2, and the ERK1/2 signaling pathway was involved in mediating ATP actions in the human reproductive system [56]. Additionally, it was demonstrated that ATP activated ERK1/2 through the P2Y2 purinoceptor/PLC/PKC signaling pathway [22]. In human endometriotic stromal cells, ATP (100 μM) increased ERK1/2 phosphorylation, which was involved in endometriosis pain [20]. MAPK ERK1/2 has a key role in the expression of proinflammatory cytokines. The inhibition of the MAPK pathway in bovine endometrial epithelia and stromal cells reduced LPS-induced IL-8, IL-6, and IL-1β expression [7,57].

The effect of suramin on cellular viability of BEND cells also was evaluated. BEND cells were incubated with vehicle (0.1% DMSO) or different concentrations of suramin (1, 10, 100, or 300 μM) for 30 min or 24 h. We observed that suramin did not reduce cell viability when compared with the vehicle (Figure 2D). Therefore, the attenuation of IL-8 production, intracellular calcium mobilization, and ERK1/2 phosphorylation produced by suramin were effects mediated through the P2Y receptors.

### 3.3. Endometrial Cells Express P2Y mRNA Receptors

**Figure 3:**
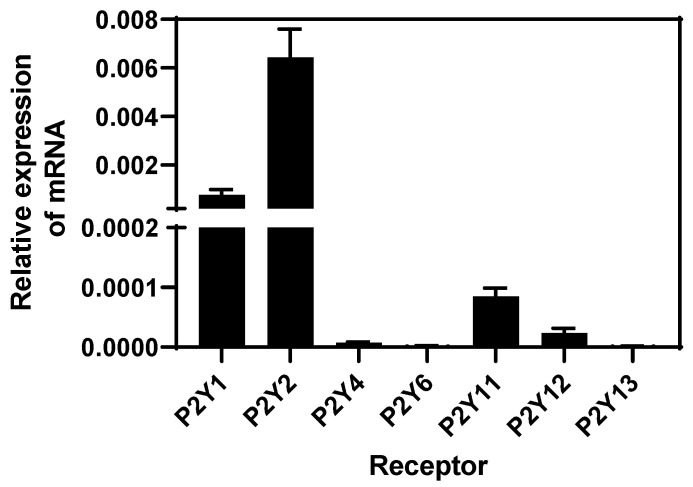
Levels of expression of mRNA P2Y subtype receptors in BEND cells. Total RNA was isolated from BEND cells and RT-qPCR for P2Y subtype receptors was performed. Level of expression of each subtype receptor was determined by the 2^−ΔΔCt^ method. n = 3 independent experiments.

Since extracellular ATP mediates its functions through binding purinergic type P2 receptors, we assessed the presence of mRNA of subtype P2Y receptors in BEND cells through RT-qPCR and analysis with the 2^−ΔΔCt^ method. BEND cells expressed higher levels of P2Y1 and P2Y2 purinergic subtype receptors, lower levels of P2Y11 and P2Y12 receptors, and P2Y4, P2Y6, and P2Y13 mRNA receptors were undetectable (Figure 3). P2Y receptors are predominantly activated by ATP or ADP, specifically P2Y-1, -2, -4, and -11 by ATP [14]. A P2Y1 receptor can be activated by ATP, which is considered a partial agonist, but ADP present highest affinity by the receptor [58]. Although the presence of P2Y receptors has been studied in human endometrial cells, in bovines, the expression has only been studied in other cell types (as mentioned in the Introduction). Human endometrial stromal cells express the P2Y2 receptor [56]. Additionally, P2Y2, P2Y14, and P2X7 are expressed in the human endometrium [21,22,23] with P2Y2 in human endometrial cancer cell lines [24], and P2Y1, P2Y2, P2Y4, and P2X7 in the rat uterine epithelium [25]. Thus, our research shows, for the first time, the presence of P2Y receptor subtypes in bovine endometrial cells. 

P2Y subtype receptors couple to different G-protein. P2Y1 and P2Y2 are receptors linked to Gαq protein, and their activation stimulates the pathway of PLC/DAG/IP3/calcium [14,16]. The increase of intracellular calcium induced by ATP in BEND cells correlates with the types of receptors expressed at a higher level. Specifically, P2Y2 receptor is expressed at the highest level compared with P2Y1. The P2Y11 receptor is considered an unconventional member of the P2Y family receptors, because it couples with both Gαq and Gαs proteins, and its activation increases IP3 and cAMP, respectively [14,16]; however the expression of the P2Y11 receptor was lower in BEND cells. The P2Y12 receptor is coupled to Gαi protein, and it is activated by ADP.

P2 subtypes receptors induce classical signaling pathways and gene expression; P2Y1 and P2Y2 activate ERK1/2 in different cell types [22,59,60,61], and increase the expression of pro-inflammatory genes such as IL-6 and IL-8 [62]. On the contrary, it has been suggested that the P2Y11 receptor has two types of responses, because it activates the pro-inflammatory NF-κB/ERK1/2 pathway and also activates anti-inflammatory signaling. Activation of P2Y11 receptor inhibited LPS-induced TNF-α production in M2 macrophages, which is proposed to occur through a regulated control of IL-1 receptors [62,63].

## 4. Conclusions

This study showed that ATP increased IL-8 production, activated intracellular calcium mobilization, and induced ERK1/2 phosphorylation in BEND cells—effects that were partially reduced by a P2Y receptor antagonist. P2Y1 and P2Y2 receptors are expressed at high levels as compared to other subtypes of receptors in BEND cells. These results suggest a key role of P2Y receptors in endometrial inflammatory activation, which could be useful as a therapeutic strategy to regulate uterine inflammation through the modulation of P2Y receptors.

## Figures and Tables

**Table 1:** Primers used in the RT-qPCR assay.

Name	Primer 5’–3’	Size	Accession #
P2Y1R	F: TTCCACATGAAGCCGTGGAG R: GGCAGAGTCAGCACGTACAA	83	NM_174410.3
P2Y2R	F: CAGTCCCCACACCCTTAGTCA R: CCGGGTATTTATGTGGGAGTCA	112	NM_001166525.1 [11]
P2Y4R	F: CCCGTGACCTATGCAGTTGT R: GCGAAAGAGGAAGAGCCAGA	75	NM_001256557.1
P2Y6R	F: GCTACTAAGGCGTGCGTTTC R: GGTTGCAGTCTACTTATCTCCCC	120	NM_001192295.1
P2Y11R	F: AGTGGCCTCCAAGATGACTTTC R: GCTCCCGGCTGCAGAAG	103	NM_001204449.1 [11]
P2Y12R	F: TACAGTCAGGCCACAAGAACA R: TGTCTTAGTTTAGCTGCTCACA	91	NM_001001174.2
P2Y13R	F: TGCCAGTAGCTCCAACACAA R: AGGGTTGAGCAAGTTCCAGG	85	NM_001098152.1
RSP9	F: GCTGACGCTGGATGAGAAAGACCC R: ATCCAGCACCCCGATACGGACG	85	NM_001101152.2 [22]

## Data Availability

The data presented in this study are available on request from the corresponding author.
